# Design, implementation, and evaluation of self-care program in the prevention of breast cancer among women in Isfahan: a community-based participatory action research protocol

**DOI:** 10.1186/s40695-022-00077-8

**Published:** 2022-07-05

**Authors:** Maryam Kianpour, Fariba Taleghani, Mahnaz Noroozi, Mitra Savabi-Esfahani, Zahra Boroumandfar, Tahereh Changiz, Zahra Ravankhah, Shaghayegh Haghjooy Javanmard, Maryam Sadat Hashemi

**Affiliations:** 1 Nursing and Midwifery Care Research Center, Department of Midwifery and Reproductive Health, Faculty of Nursing and Midwifery, Isfahan University of Medical Sciences, Isfahan, Iran; 2 Nursing Midwifery Care Research Center, Faculty of Nursing Midwifery, Isfahan University of Medical Sciences, Isfahan, Iran; 3 Department of Midwifery and Reproductive Health, School of Nursing and Midwifery, Isfahan University of Medical Sciences, Isfahan, Iran; 4 Department of Medical Education, Educational Development Center, Medical Education Research Canter, Isfahan University of Medical Sciences, Isfahan, Iran; 5 Isfahan Cancer Registry, Isfahan University of Medical Sciences, Isfahan, Iran; 6 Department of Physiology, School of Medicine Applied Physiology Research Center, Cardiovascular Research Institute, Isfahan University of Medical Sciences, Isfahan, Iran; 7 Nursing and Midwifery Care Research Center Department of critical care, Faculty of Nursing and Midwifery, Isfahan University of Medical Sciences, Isfahan, Iran

**Keywords:** Breast cancer, Prevention, Action research, Self-care

## Abstract

**Introduction:**

Breast cancer is one of the most prevalent cancers among women in Isfahan, Iran; however, its prevention is not desirable in this city. This disease poses several health, social and economic challenges for women. To promote women's self-care in breast cancer prevention, this study aims to design, implement and evaluate a self-care program among women in Isfahan through using a community-based participatory action research method.

**Methods:**

The present study is based on a community-based participatory action research approach. In this study, the participatory action research includes four general phases of organizing, action planning, action, and rethinking. These phases are summarized as follows: In the organizing phase, the needs of the participants and the action research settings are examined. This means that the current situation is identified and the views of the process owners are assessed. In the action planning phase, using the results of the first phase, some strategies are designed to promote self-care behaviors in the prevention of breast cancer among women in Isfahan. In the implementation phase, the selected strategies are implemented with the help of the process owners. Finally, in the rethinking phase, the results of the implementation of the strategies are monitored and evaluated. This cycle continues until the intended results are achieved.

**Discussion:**

Changing the role of individuals from a passive status to an aware and active status in the care process requires motivation, responsibility, and active participation of individuals in the disease control process. Moreover, many cultural and social factors affect the active participation of Iranian women. Therefore, individuals can be involved in promoting their health using a community-based participatory action research approach.

**Supplementary Information:**

The online version contains supplementary material available at 10.1186/s40695-022-00077-8.

## Introduction

According to previous studies, breast cancer is one of the most prevalent cancers among women in Isfahan. Breast cancer can lead to health, social, and economic challenges for women. A community-based participatory action research can help individuals in promote their health. This participatory action research is conducted in four phases: organizing, action planning, action, and rethinking [1–5].

## Background

Breast cancer is the most common cancer and is the second leading cause of cancer death among Iranian women and the western world. The incidence of this disease is increasing rapidly as more than two million new breast cancers were diagnosed in 2018 [6]. Given the steady increase in the incidence of the disease, it is predicted that 2.7 million new cases will be diagnosed by 2030, 60% of which (1.72 million) will be in less developed countries [7]. In other words, the burden of the disease is expected to increase in developing countries in the future [6].

The epidemiological pattern of breast cancer in Iran is similar to it in the Eastern Mediterranean and other developing countries. This cancer has been declared as the most common cancer and the most common cause of death among Iranian women. The incidence of this cancer has been changed in recent years as the number of patients with breast cancer is increasing [8]. According to researchers of a study conducted in Isfahan, breast cancer breast cancer is one of the most prevalent cancers among women in Isfahan [9]. However, in this city, the prevention of breast cancer is undesirable and many women are referred to the medical centers in the advanced stages of the disease [9, 10]. Cancer imposes a heavy economic burden on the patient and the health system. The cost of treatment is much higher per person than the cost of prevention because of limited financial and human resources [10, 11]. Indirect costs of breast cancer including medical visits, decreased income from work absences, and reduced job benefits are estimated to be $ 11,527 per patient [12]. Moreover, following cancer and its treatment, patients suffer from many physical and psychological complications that make them unable to play their roles properly in family and society. Additionally, breast cancer can challenge women's sexual identity as the breast is part of female’s identity [13, 14]. Many studies have indicated that women with breast cancer have a higher rate of sexual dysfunction (impaired sexual desire, orgasm disorder, and sexual arousal disorder) [15–27] and a weaker body image than healthy women. Some breast cancer treatments, including mastectomy, significantly reduce sexual arousal, increase dyspareunia, and prevent orgasm [28–30]. Mastectomy had a significant effect on couples' sexual satisfaction compared to lumpectomy, as after mastectomy, individuals had sexual dysfunction. Many women experience stress, anxiety, and depression after mastectomy, affecting their sexual function. Major issues and problems that typically affect the quality of life and sexual function of cancer patients include the psychological and emotional effects of the disease and diagnostic and therapeutic measures. These psychological stresses and the behaviour of caregivers are factors that affect the sexual function of cancer patients. According to a study conducted by Khan Babaei, sexual violence in improved breast cancer patients after mastectomy was moderate but humiliation of the spouse during sexual intercourse had the highest score [30]. On the other hand, chemotherapy affects women's sexual function and marital relations due to nausea and vomiting, weakness, vaginal dryness (leading to dyspareunia and delayed orgasm). These patients develop premature menopause. Moreover, decrease in estrogen after chemotherapy leads to vaginal atrophy. Additionally, decrease in androgen reduces women's sexual desire and arousal [24].

As it is mentioned, breast cancer can bring about many health, social, and economic challenges for women. However, through designing effective interventions for the promotion of self-care behaviors, this disease can be controlled a prevented. In this regard, Isfahan University of Medical Sciences, as the custodian of health in Isfahan province, intends to reduce the incidence of breast cancer in Isfahan by promoting health and self-care behaviors in women.

The core principle of the new public health movement is the recognition of the need for tackling the wider determinants of health the social and environmental factors. Empowerment, community participation and capacity building are increasingly seen as strategies for achieving systemic change [31].

Changing the role of individuals from a passive status to an aware and active status requires motivation, responsibility, and active participation of community members in the disease control process. According to Health Belief Model, people must believe that even in the absence of any symptom, the disease may exist. When people find themselves at risk of the disease (perceived susceptibility) and realize that the disease has serious potential consequences (perceived seriousness) and believe that prevention would have positive results (perceived benefits) and barriers of that behavior are fewer than obtained benefits (perceived barriers) and believe that they have the ability to perform health behavior activities (self-efficacy), it would be more probable for them to accomplish this behavior [32].In this regard, the research team intends to reduce the incidence of breast cancer in Isfahan by promoting women's health and self-care behaviors through using a community-based participatory action research method.

### The research objectives

1. To determine the risk factors for breast cancer in women
2. To explain barriers and facilitators for self-care in breast cancer prevention among women in Isfahan
3. To design a self-care program for the early identification and screening of breast cancer among women in Isfahan
4. To explain the effects of the implemented program on self-care behaviors for the prevention of breast cancer among women in Isfahan

## Research design and methods

The present study is based on a community-based participatory action research approach. Participatory action research is the most appropriate approach for the implementation of change. This kind of research is methodologically flexible and depends on the research setting. Action or change is the core of the process and the members of the research team participate equally in the process and finally, the decision for the implementation of operations depends on the participants. The researcher facilitates but does not control the process. Action research has a cyclical nature that begins with the identification of the problem and continues with action planning and, then, with the evaluation of the actions and obtained results [33].

This participatory action research is conducted in the four phases of organizing, action planning, action, and rethinking. These phases are summarized as follows:

In the organizing phase, the needs of the participants and the action research settings are examined. This means that the current situation is identified and the views of the process owners are examined.

In the action planning phase, using the results of the first phase, strategies for the promotion of self-care behaviors are designed in order to prevent breast cancer among women in Isfahan.

In the action phase, the selected strategies are implemented with the help of the process owners.

In the rethinking phase, the results of the implementation of the strategies are monitored and evaluated, and this cycle continues until the optimal results are achieved [31].

## Phase 1: Organizing

### The objectives of this phase are as follows:

1. To determine the risk factors of breast cancer in women
2. To explain barriers and facilitators of self-care in breast cancer prevention among women in Isfahan

In this phase, the following actions are taken to achieve the objectives:

1. Review of the studies conducted in Iran and the world to evaluate the risk factors for breast cancer in women as well as the self-care promotion strategies for preventing breast cancer
2. Specify the research settings and process owners
3. Identify committees and existing support units in the study settings
4. Negotiate with the process owners
5. Identify the barriers and facilitators of the research implementation with the help of the process owners and strategies to promote self-care behaviors in the prevention of breast cancer among women in Isfahan
6. Form special committees to expedite action research processes

### Review of the literature

In this stage, in order to obtain the existing knowledge in the field of breast cancer risk factors, barriers to self-care, and strategies for the promotion of self-care to prevent breast cancer, studies conducted in Iran and the world will be reviewed. To search the existing databases, keywords are determined based on Mesh and the title of the research and, then, by combining the existing keywords, the resources will be reviewed. To this end, the keywords such as breast cancer, breast neoplasms, self-care, risk factors and prevention, breast cancer risk factors, and self-care promotion strategies in breast cancer will be searched in the databases for publishing care guidelines, GIN, NGC, NICE, SIGN, GAC, New Zealand, NHMRC, WHO, Breast Cancer Screening Guidelines such as American Cancer Society, American Cancer Society Prevention, Early Detection Guidelines, PUBMED/MEDLINE, CINHAL, COCHRANE, SCOPUS, ProQuest, IranMedex, Magiran, SID and IranDoc.

Self-care promotion strategies for the prevention of breast cancer in women are included in the decision matrices and used in the program development process.

The full text of all sources that were published in English and Persian between 2000 and 2020 are reviewed. Related articles are extracted from each database. Review studies are included in the final analysis and their sources are reviewed through cross reference method. The keywords are updated during the search, and with those keywords, the articles that are found to be relevant are searched again. The search process is performed by two information retrieval specialists and a project partner. Then, the results of their search are compared with each other every week. In all articles, hand search also perform. To find Gray Literature, resources such as http://www.gateway.com/worldwidehttp://www.proquest.com/, http://www.trialscentral.com/, http://www.irct.ir and other related websites are reviewed. The number of related articles extracted and finalized from each database is reported in EndNote, and then the flowchart of the number of articles is set. In the first stage, by quickly reviewing the titles and abstracts of the articles, a number of unrelated and duplicate studies are removed under the supervision of the responsible author. In the next step, the remaining articles carefully evaluate and critique All steps are performed by two independent researchers (Dr. Hashemi and Dr. Kianpour) and a third researcher (Dr. Taleghani) who supervise the review results of the two reviewers and in case of disagreement in deleting or accepting articles, her decision is the base.

#### Identification of the Study Settings

##### Study Setting

To interview employed and non-employed women in the Isfahan metropolis, the researchers will refer to health centers, offices, cultural centers, mosques, parks, recreation centers, clubs, and homes. In this study, participants are selected by purposive sampling. Hence, service providers, clients, and policy makers who meet the criteria for entering the research and are good sources of information are selected. For a deeper understanding of the barriers and strategies for promoting self-care behaviours, two groups of help-seekers who live in 15 districts of Isfahan (with a history of screening procedures and not performing screening procedures and referring to health centres in Isfahan) are selected for group discussion. Because self-care behaviours are influenced by demographic characteristics (age, marital status, level of education, income, occupation, ethnicity, and place of residence), maximum diversity is considered in terms of these characteristics. The location of the potential participants is also taken into account when selecting the samples. Participants are selected from 15 districts of Isfahan. The socio-economic level and the level of access to health services in the residents of different areas of Isfahan are different from each other. In the first group discussion, participants are purposefully selected from women who have a health record and have undergone screening procedures. Snowball sampling method is used to identify participants who do not have good self-care behaviours and do not go to health centres for self-care training and screening. In this method, the first participants introduce other people to participate in the study. The snowball sampling method is used when research participants are outside the health care system and are not easily identifiable. In order to identify these women, social activists from each neighbourhood are used. With the cooperation of community activists in each neighbourhood, women who do not participate in self-care and screening programs are identified, and then these individuals introduce other participants to the researcher. In order to interview with working women, the type of job that may affect the level of awareness, health beliefs, and access to health services is considered. Thus, the maximum variety of jobs (housewives, workers, faculty members, teachers, team members Health, nurses, midwives, doctors, service personnel, government, and non-government administration staff in Isfahan) are taken into account during sampling. For this purpose, correspondence is made with the managers of the above-mentioned departments and organizations, and a list of working women who are aged 20 to 69 is provided to the research team. Moreover, the number of samples is calculated as a quota according to the number of women who works in each organization and then sampling is done. To conduct the interview, the researchers will refer to the offices of the experts and specialists in hospitals, universities, Isfahan health center, and sonography and mammography units.

##### Study population

In community-based action research studies, the views of those persons who are at risk should be evaluated. The combination of community members’ experiences with public health science provides a deeper understanding of complex social phenomena Thus, providing more relevant interventions and increasing the likelihood of the interventions can be effective. They can also be adopted, implemented, and sustained in a real-world setting [34].

Accordingly, employed and non-employed women who live in Isfahan (15 districts) are part of the study population. Moreover, explaining the views of the process owners is of great significance to design strategies for promoting self-care behaviors in the prevention of breast cancer among women in Isfahan. As such, service providers (gynecologists, radiologists, general surgeons specializing in breast surgery, specialists in reproductive health, general practitioners, nurses and midwives, health policymakers, officials and experts of the Cancer Department of the Ministry of Health, experts of the Non-Communicable Diseases Control Center and the middle-aged unit of the provincial health center) are also among the study population.

#### Identification of the process owners

As the purpose of this study is to promote self-care behaviors in the prevention of breast cancer among women, the process owners of this study are as follows:

• Both employed and unemployed women living in Isfahan
• Policymakers, officials, and experts of the Cancer Department of the Ministry of Health
• Experts of the Cancer Department of Isfahan Health Department
• Middle-aged Unit of Isfahan Health Center (Middle-aged people health unit plays a role in preventing non-communicable diseases in Isfahan province. This unit operates under the supervision of Isfahan University of Medical Sciences. It attempts to increase life expectancy, reduce the burden of disease and risk factors, and offer public health services with an emphasis on primary care)
• Faculty members of Oncology Departments, gynecologists, general surgeons specializing in cancer surgery, adult health nursing, community health nursing, midwifery, and reproductive health in the School of Nursing and Midwifery of Isfahan University of Medical Sciences

### Identification of the committees and support units in the study settings

The committees and support units in this study are as follows:

• Vice-Chancellor of Research in Isfahan University of Medical Sciences
• Cancer Department in the Isfahan Health Department
• Middle-aged Unit of Isfahan Health Center
• Isfahan Municipality Health Culture Committee
• Isfahan Health Donors Association
• Active NGOs in the field of health in Isfahan province

### Negotiation with process owners

In this study, self-care packages and educational media are developed with the help of the faculty members of Isfahan University of Medical Sciences develop.In the present study, the design of a self-care education program to prevent breast cancer is based on the ADDIE^1^ model [31].In the first step, the content, platforms, and media required for self-care education are extracted. In the second step, the program is designed based on the findings of the analysis in the previous step, learning objectives, sequence of objectives, and educational and assessment strategies. Then, it will be provided to experts for approval. In the third step, a learners' guide is prepared, a pilot study is conducted, and formative reviews (using the women’s opinions in Isfahan and experts) are conducted. In the fourth step, the program is made available to learners.

The necessary negotiations will also be held with the Health Culture Committee of Isfahan Municipality, the active NGOs in the field of health in Isfahan province, and the Secretariat of Cultural and Artistic Centers of Isfahan Mosques, to hold health campaigns in parks, recreation centers, and mosques of Isfahan. The research proposal will be sent to the Vice-Chancellor of Research at Isfahan University of Medical Sciences for financial support. Moreover, for further funding support, the research proposal will be sent to the World Health Organization in the Ministry of Health and Medical Education, Isfahan Municipality Health Culture Committee, Isfahan Health Donors Association, and active NGOs in the field of health in Isfahan province. Additionally, to promote women's health awareness of breast cancer, the necessary consultations will be carried out with the Isfahan broadcasting organization.

### Identification of the barriers and facilitators of the research implementation

This qualitative research is used a content analysis approach.

#### Participants

In the present study, the participants will be selected using purposive sampling method. After obtaining the necessary permits by visiting the above-mentioned centers in-person, the experts who meet the inclusion criteria in the field of breast cancer prevention will be selected and interviewed after obtaining their informed and written consent. The researcher continues to select and interview with participants until data saturation is reached. The interviews continue until the interviews do not add any new data to the previous ones. In qualitative research, the number of participants is determined during the research, and the participants are selected using non-random and a purposive method which may be based on the inclusion criteria. Therefore, there is no need in these studies to estimate the number of. participants in advance, and the purposive sampling method is used [34].

Also Snowball sampling method is used to identify participants who do not have good self-care behaviors and do not go to health centers for self-care training and screening. In this method, the first participants introduce other people to participate in the study. The snowball sampling method is used when research participants are outside the health care system and are not easily identifiable. In order to identify these women, social activists from each neighborhood are used. With the cooperation of social activists in each neighborhood, female community liaisons, and charities, all women are identified in each neighborhood, and women who do not participate in self-care and screening-training programs are also identified. These people then introduce the other participants to the researcher. In this study, to ensure that the sample represents the community, the maximum diversity in sampling (age, marital status, level of education, income, occupation, ethnicity and place of residence) is observed [35].

In qualitative research, unlike quantitative research, instead of the number of samples, the quality of the data is emphasized. In qualitative research, the sampling process continues to the point where the researcher does not receive new information from the participants, and only the data should be repeated and verified, even though there is no fixed standard and rule for the number of participants in this kind of research. Given time and cost constraints and initial targeted sample size is usually proposed (eg 30) [36].

#### Inclusion criteria

In Iran, according to the guidelines for screening and prevention of breast cancer, women between the ages of 20 and 69 are examined by a midwife or a trained doctor in health centres. Moreover, the necessary self-care training, encouragement of monthly self-examination, clinical examination, annual and ultrasound, and mammography are done in case of necessity. In the current study, all individuals (both male and female) who are involved in cancer prevention and treatment will be interviewed. These individuals include men whose spouses do not participate in women's self-care and screening programs, insurance agents, male surgeons, and health care assistants at the University of Medical Sciences Isfahan.

The participants of this study are employed and non-employed women in Isfahan, gynecologists, radiologists, general surgeons specializing in breast surgery, specialist in reproductive health, nursing, and health education. Moreover, among other participants are health policymakers, experts of the Cancer Department of the Ministry of Health, experts of the Non-Communicable Diseases Control Center and the middle-aged unit of the provincial health center, general practitioners, midwives, and nurses who have experience in educating, treating and caring for breast cancer patients.

One of the conditions for entering the study is to have an informed consent. It means that participants have sufficient information about the research and are able to understand the information. It also signifies that they have the right to choose freely to participate or not to participate in the study. Thus, people with mental health problems are not included in the study. Participants are given sufficient and necessary information about the types of questions that are asked, how to use the results, and their anonymity in all stages of the research. One of the important points in obtaining consent is that consent must be completed consciously and voluntarily, in other words, participants must have sufficient information in the field of research and be able to understand the information and also have the free choice to participate or not to in the research. Since some participants may be illiterate, the consent form is read to them by the researcher and it is ensured that the participant understands the purpose of the study, then, the form is completed and signed by the person's guardian.

### Exclusion criteria

Every participant who is not willing to continue his/her cooperation can be excluded from the study.

#### Data collection

The data collection method at this phase includes open and semi-structured interviews and focus group discussions (FGDs).

Focus group interview aims at collecting high-quality data in a social context. This primarily helps understand a specific problem from the viewpoint of the research participants. The questions about why and when focus group interview should be conducted is very significant. Firstly, focus groups provide a rich and detailed set of data about perceptions, thoughts, feelings and impressions of people in their own words. Secondly, focus groups are predominantly beneficial when a researcher intends to find out the people ‘s understanding and experiences about the issue and reasons behind their particular pattern of thought. Thirdly, this method is suitable for examining sensitive issues (e.g. breast cancer screening and obtaining information from very sensitive population). Fourthly, use of focus groups gives an opportunity to marginalized segments of society (e.g. minorities, women, and people of color) for expressing their feelings about their needs and problems [37].

There are two focus groups in the current study. In the first focus group, participants are purposefully selected from women who have a health record and have undergone screening procedures. Snowball sampling method is used to identify participants who do not have good self-care behaviours and do not go to health centres for self-care and screening. In this method, the first participants introduce other people to participate in the study. The snowball sampling method is used when research participants are outside the health care system and are not easily identifiable. In order to identify these women, social activists from each neighbourhood are used. With the cooperation of social activists in each neighbourhood, women who do not participate in self-care and screening programs are identified. Then these people introduce other participants to the researcher. All people will be contacted and after obtaining the informed consent, the second focus group will be implemented. Group discussion questions are similar to individual interview questions. The duration of each group session is between 1.5 to 2 h and the number of people who participate in these sessions is between 5 and 12. In order to standardize the interviews, all researchers who are involved in data collection receive the necessary training on how to observe ethics and conduct the interviews. In order to protect the rights of the participants, the researchers contact the participants in person before the interview sessions. Then, they introduce themselves and the objectives of the research. After that the time and place of the interview will be determined by the participants. All participants take a taxi to travel and participate in the focus group. The transportation costs are paid by the research team.

In the present study, employed and non-employed women of Isfahan metropolis and members of the health group who are eligible are selected using the purposive sampling method. Working women have higher income levels than unemployed women. Therefore, they usually have higher independence of action and their access to public services is higher. The type of job may affect the level of awareness, health beliefs, and access to health services is considered. Thus, the maximum variety of jobs (housewives, workers, faculty members, teachers, health team members, nurses, midwives, doctors, service personnel, government, and non-government administration staff in Isfahan) are taken into account during sampling.They are invited for in-depth, semi-structured, and individual interviews after obtaining their informed consent. In semi-structured interviews, there are no fixed, pre-determined questions, and the questions are formed based on the interview process. The followings are some sample questions asked the participants:

• Have you heard about self-care and breast exams?
• What is your experience in breast screening?
• Why do not some women attend breast screening?
- Sample questions asked of health team members are as follows:
• Please tell us about your experiences of breast cancer prevention among women in Isfahan?
• What are the barriers and facilitators in preventing breast cancer among women in Isfahan?
• In your opinion, what measures should policymakers take to reduce the risk factors for breast cancer among women in Isfahan?
• What strategies do you recommend in order to remove barriers to modulating breast cancer risk factors among women in Isfahan?

The questions are then altered in the aim to reach saturation. The duration of each interview depends on the situation and environmental conditions, the agreement of the parties, the subject of the interview, and the used method. In this study; the duration of the interviews is from 30 to 60 min. This period depends on the participants' circumstances, their willingness to continue the interview, as well as the time to obtain in-depth data in answering the interview questions. In this regard, Polit and Beck (2014) state that the place and time of the interview are at the disposal of the interviewee, but usually different sources suggest between 30 and 60 min for the interview (39) [38]. In order to encourage the participants, gift cards of 20,000 Tomans will be given to them. The research participants take a taxi to travel and their transportation costs are paid from the project budget.

Interview and sampling continue until data saturation is reached. Saturation refers to the completion of all categories and to the idea that no new conceptual information that requires a new code or the expansion of new code is obtained..

At the group discussion sessions, the researcher acts as the facilitator and guider of the discussions, and another person is present to take notes. In the present study, the researcher plays the role of the facilitator in focus groups. The role of a facilitator or a moderator is very important in conducting a focused group research. Therefore, it is very important to invite experienced researchers to do such an activity. In the present study, Dr. Savabi is responsible for mediation due to her familiarity and mastery of the subject and breast cancer screening. Also, since focus group is a type of semi-structured group interview, a comprehensive guide is provided to conduct the discussion. This guide is used as a roadmap for group interviews and specifies what goals should be achieved after the group interview.

At one end of the spectrum, the mediator plays the role of a co-creator, playing a more active role and intervening more in the debate, while at the other end of the spectrum, the mediator acts only as a data collector and raises only broad and neutral questions and minimizes its role in the debate and intervenes only where it ends leads to data production.

#### Data analysis method in the qualitative phase

Investigators will use content analysis in this qualitative research project [35]. In this research, in order to analyse qualitative data, the conventional qualitative content analysis method with Granheim and Landman’s approach is used. First, interviews are copied word-by-word. Then, in order to be familiar with the text, the researcher reads the text of the interviews several times and writes down his / her general impression of them. In the next step, the important sentences are underlined and the semantic units are identified. Then, relevant codes are assigned to them. In the next step, continuous comparison analysis method is used to determine the subclasses and classes. In this way, new data are constantly compared with previous data in terms of similarities and differences, and extracted codes that have similarities are placed in a subclass. Classes are then created by grouping sub-classes. Continuous comparative analysis is used to determine the classes. In this way, subclasses that have a common feature are placed in one class. There is the most homogeneity within classes and the most heterogeneity among classes [36].

#### Phase 2: Action planning

The purpose of this phase is to design a self-care program for the prevention of breast cancer among women in Isfahan. To achieve this purpose, the following activities are performed in this phase:

1. Determining criteria for prioritizing strategies
2. Review of the strategies in group discussions with the presence of executive committee members and process owners
3. Final review and approval of strategies in the Joint Committee
4. Planning for the implementation of the strategies in the study setting (development of operational plan)

### Section 1.2: Examining the strategies in group discussions with the presence of the executive committee members and the committee of the process owners

For the prioritization of the strategies, an expert panel is formed. In this phase, the strategies obtained in the organizing phase, which is the result of the literature review and qualitative content analysis (semi-structured interviews and FGDs with women and semi-structured interviews with process owners), will be used to develop the program. Since it is not possible to implement all the strategies obtained from the organizing phase, the proposed strategies should be prioritized. Decision matrices will be developed to prioritize the extracted strategies. Self-care promotion strategies for breast cancer prevention are put in the rows of decision-making matrices, and the criteria for prioritizing strategies (ease of implementation, cost-effectiveness, time-consuming, effectiveness, efficiency, acceptability, and compliance with policies and values of the community) are put in its columns. Then, the members of the expert panel are asked to give each strategy a score of 1 to 5. Then, the mean score of the available strategies is determined and the program is developed based on the obtained scores.

The matrices are distributed among process owners and experts in the field of breast cancer prevention. After collecting the matrices, the researcher enters the data into SPSS software version 16 and calculates the mean and standard deviation of each item using the basics of descriptive statistics. The matrix items are then sorted based on the mean scores, and the priority of the proposed strategies and the items agreed upon by the experts are specified. The mean scores of each strategy and the level of the agreement are determined based on the variance of the answers and the quartile range [39] (Table1). If more than 80% of the members agree on an area and the quartile range is zero, the agreement is estimated to be very high. If more than 60% of the members agree and the quartile range is more than 1, the agreement is considered moderate; and if less than 60% of the members agree and the quartile range is more than 2, the agreement is considered weak. The consensus is reached when the level of agreement is very high. Therefore, in decision matrices, when more than 80% of members agree, consensus is reached and the strategies are introduced as the suggested ones for self-care behaviors in preventing breast cancer [39].

**Table 1:** Strategy matrix

Self-care promotion strategies to prevent breast cancer	Ease of implementation	Cost-effectiveness	Time-consuming	Effectiveness	Acceptability and compliance with organizational policies and values	total Score

This panel is formed with the presence of representatives of the employed and unemployed women and the members of the executive committee. Another panel is formed with the presence of the process owners and specialists (e.g. gynecologists, radiologists, general surgeons specializing in breast surgery, specialists in reproductive health, and nursing, general practitioners, oncology nurses, Midwives, policymakers, officials, and experts of the Cancer Department of the Ministry of Health, experts of the Non-Communicable Diseases Control Center and the middle-aged unit of the provincial health center, and faculty members of the Oncology Department). This expert panel is held in the presence of the research group members. In order to confirm the prioritization of self-care promotion strategies for breast cancer prevention, expert panels are held. Then, based on the results of the panels, the initial version of the self-care promotion program for breast cancer prevention is prepared and designed. For holding these panels, after the formal and written invitation of the intended subjects and their presence, the goals and agenda of the meetings are explained at the beginning of the meetings and, then, the researcher presents the results of prioritizing strategies for the promotion of self-care behaviors in preventing breast cancer. Then, the participants express their views on the prioritizing strategies, and have a discussion to reach a consensus. The researcher, as the secretary of the session, takes notes and records the contents of the meeting. These sessions are managed by the researcher who records all agreed and disagreed contents.

## Phase 3: Action

For better implementation of the program, activities, subjects, resources, and implementation time of each activity in the program are determined and, then, a meeting is held to prepare the executive committee members. It should be noted that in the implementation phase, the proposed measures will be taken based on the conditions, budget, time, and facilities for 25% of women (50,000 women) in Isfahan. According to the statistical yearbook of Isfahan province, the population of women who are aged 20 to 59 is 1378000 in Isfahan. The programs will be organized according to the conditions and facilities after preparation and necessary coordination with the relevant health centres, socio-cultural centres, mosques, universities, and government offices. For this purpose, necessary arrangements are done with relevant officials including: General Director of Culture and Guidance of Isfahan, Secretary of the Coordination and Supervision of Cultural and Artistic Centres of Mosques in Isfahan Province, Head of Health-Culture Development Department of Isfahan Municipality, General Directorate of Education, Head of Isfahan Radio and Television, Isfahan governor's office, and other offices in Isfahan. It is predicted that there is a need to train instructors to teach self-care topics and perform breast screening. Additionally, guidelines and instructions (including pamphlets, booklets, slides, and videos) on self-care of breast cancer prevention should be made available to executive committee members who are to provide community-based self-care training. The content of these guidelines will be provided by faculty members. Implementing the program at the level of offices, socio-cultural centres and health-friendly mosques, girls' schools, and universities requires the employment of a large number of instructors, doctors, nurses, and midwives. For this purpose, there is a call for cooperation. The hired instructors must have the necessary competencies to teach self-care topics in the field of breast cancer prevention and breast examination, so they require attending workshops and training sessions organized by the executive committee.

## Phase 4: Rethinking

This phase aims to explain the effect of the executive program on self-care behaviors in the prevention of breast cancer among women in Isfahan. Rethinking will be performed in this phase. Rethinking is a mental process in which events, experiences, problems, or situations are re-examined so that a better understanding of the situation can be achieved. This understanding will lead to the appearance of strategies, ideas, improvements, and changes. Rethinking is, in fact, a dialogue of thought enabling the researcher to determine the emerging patterns of interactions between participants and the environment. It asks what strategies are effective, what changes are needed in the environment, what solutions are effective, what changes in the environment are needed, what changes are vital, what other information we need, and what action plans are required. In this research, rethinking is done in two stages: 1) During the implementation stage, and 2) In the final stage. Each stage will be discussed in detail.

### Rethinking during the implementation stage of the program

In order to identify issues and problems during the implementation of the program, feedback is taken from the participants including women, who participate in the study, members of the research team, and the executive committee. In this study, for rethinking during the implementation, the Gibbs' framework which has six stages is used [37].

In the first stage, the event which should be reconsidered (women's self-care behavior promotion program) is described in full details (the setting and context of the event, the outcome, the people involved in the event, and how it is implemented). The second stage is to examine the feelings and thoughts existing in the minds of the research team members, the executive team and the participating women. To this end, the following questions are used: How did you feel when the program started? What were you thinking about at the time? And how did the program make you feel? What feelings are created in you by the other team members? How do you feel about the outcome of the program? Then, in the third phase, assessment is performed. In the fourth stage, the program for promoting women's self-care behaviors about breast cancer prevention is broken down into its components and each component is examined separately. This stage examines, what has been performed well? What has been wrong or has not changed and what has been conducted well? What have others done well? And what should be done for the better implementation of the program? The conclusion is made in the fifth stage. In this stage, the opportunity is provided for learning from previous experiences based on the analysis of the events in the previous stages. In the sixth stage, planning is done for the next cycle. In other words, it is examined whether it will be done differently or similarly in the future? [38].

Various methods of data collection such as self-report, field notes, and review sessions are used to get feedback during the implementation of this research.

A) Self-report technique: Self-report techniques are the most prevalent methods of data collection in clinical studies. These techniques are very powerful, due to their immediacy. Using these techniques, researchers usually obtain information that is difficult to gain through other methods.
B) Field notes: researchers observe women's self-care behaviors in different situations and times, and during different stages of the study. In this method, necessary notes are taken.
C) Review sessions: Holding regular meetings by the research team from the beginning of the study to its final stages is one of the effective and efficient ways to obtain the opinions, views, suggestions, and criticisms of the participants. These meetings are held every two weeks after each intervention.

### Final assessment of the program (final rethinking)

Quantitative and qualitative methods are used for the final assessment of the program. To evaluate the quality of the study, the participants' experiences about the effectiveness of the program are considered after the program. Moreover, after the implementation of the program, the level of the knowledge, attitude, and practice of women about self-care behaviors for preventing breast cancer is evaluated through a questionnaire to determine the effectiveness of the program. Moreover, quantitative indicators such as the number of patients who referred to the medical centers and doctors' offices for ultrasound, mammography, or breast examination are used to determine the effectiveness of the program.

## Discussion

Numerous studies have considered the role of socio-cultural factors in women's self-care behaviors. Lack of understanding and underestimating the risks and complications of breast cancer affects women's participation in self-care programs [39, 40]. In some cultures, women believe that what is not paid attention to will not happen or will eventually disappear. Such beliefs are rooted in the cultural issues of society and may affect the extent to which women participate in self-care programs and the prevention and control of breast cancer. Health belief model constructs consist of perceived sensitivity, perceived intensity, perceived benefits, perceived barriers, self-efficacy, and guidance for action. According to the Health Belief Model, in order to perform a healthy behavior, individuals must first feel threatened by the problem (perceived sensitivity). Then, the depth of the risk and the severity of its complications should be considered (perceived severity). Finally, individuals should believe in the usefulness and applicability of the healthy behavior based on the positive feedbacks they receive from the environment (action guide). According to this model, individuals are convinced to carry out disease prevention activities with positive feedback they receive from their environment (ease of doing, being time consuming and also the effective role of self-examination in early diagnosis and treatment of the disease as well as preventing the spread of the disease to other parts of the body and being cost-effectiveness Therefore, in educating women, they should be sensitized about the risk of breast cancer. Moreover, the norms of society about self-care behaviors should be considered [41]. In Iranian society, fear of cancer diagnosis [42], lack of trust of health lack of women’s independence in decision-making, fatefulness, lack of women's empowerment [43], and personality traits (not paying attention to one's health and prioritizing family, as well as emotional responses such as the conflicting effects of fear and shame) are the most important obstacles to women's participation in self-care programs [44, 45]. In order to remove these obstacles, the design of the prevention programs according to the economic, social, and cultural conditions of the Iranian society seems to be necessary.

The most important limitation in conducting a study is covid-19 pandemic. Many participants may be reluctant to participate in a research project in order to comply with health protocols and fear of being in crowded places due to corona virus pandemic.

## Conclusion

The self-care program in the prevention of breast cancer among women in Isfahan is a missing link in the provision of services related to women's health which can play an important role in the promotion of health and the prevention of the disease. In particular, the age of breast cancer in Iranian women is a decade lower than the average age of it in the world. Changing the role of individuals from a passive status to an aware and active one in the care process requires motivation, responsibility, and active participation of people in the disease control process. Since various socio-cultural factors affect women's self-care behaviors, the design of prevention programs based on socio-cultural conditions and the participation of citizens has a special priority to provide more optimal services.

Therefore, it seems that using a community-based participatory action research, women can be involved in promoting their health. The study’s findings may help public health educators,

Health promoters, social workers, and policy makers to understand the critical role of effective, culturally based preventive strategies as well as women’s needs. This study also provides some insights into the health behavior factors that need to be considered if effective strategies and intervention programs are to be designed to promote women’s health and, subsequently, the health of their families in Iran and those of other women who may have similar beliefs and practices that need to be dealt with effectively.

## Supplementary Information

**Additional file 1**.

## Data Availability

Not applicable.

## Abbreviation

FGDs: Focus group discussions

## Footnotes

1 Analysis, Design, Development, Implementation, Evaluation (ADDIE).
