# Supplementary material for: Design, implementation, and evaluation of self-care program in the prevention of breast cancer among women in Isfahan: a community-based participatory action research protocol
Source: Womens Midlife Health. 2022 Jul 5;8:7. doi: 10.1186/s40695-022-00077-8 (PMC9254568; doi:10.1186/s40695-022-00077-8)
Supplement: Supplementary file 1 — Additional file 1. [file 40695_2022_77_MOESM1_ESM.docx]

| Section | Question | Answer |
| --- | --- | --- |
| Background | 1. p. 2. line 51 many women self-refer? or are referred? | Sentence was corrected.  However, in this city, the prevention of breast cancer is undesirable and many women are referred to the medical canters in the advanced stages of the disease |
|  | 2. p. 3 line 22 - as THE breast is part ... | Sentence was corrected.  Moreover, following cancer and its treatment, patients suffer from many physical and psychological complications that make them unable to play their roles properly in family and society. Additionally, breast cancer can challenge women's sexual identity as the breast is part of female’s identity |
|  | 3. p. 4. l;ine 27 barriers of that behavior ARE fewer | Sentence was corrected.  According to Health Belief Model, people must believe that even in the absence of any symptom, the disease may exist. When people find themselves at risk of the disease (perceived susceptibility) and realize that the disease has serious potential consequences (perceived seriousness) and believe that prevention would have positive results (perceived benefits) and barriers of that behavior are fewer than obtained benefits (perceived barriers) and believe that they have the ability to perform health behavior activities (self-efficacy), it would be more probable for them to accomplish this behavior |
|  | p. 5 You use the phrase preventing breast cancer, but do you really mean EARLY IDENTIFICATION or SCREENING or EARLY DETECTION? | Sentence was corrected.  To design a self-care program for the early identification and screening of breast cancer among women in Isfahan |
| RESEARCH DESIGN AND METHODS: | p. 6 line 48 Do you mean "a hand search was also conducted of references cited in selected publications". I found the sentence in line 28 unclear. Line 51 The number of related articles extracted and fainlized from each database is reported in ENdnote . | Sentence was corrected.  The number of related articles extracted and finalized from each database is reported in EndNote, |
|  | p. 7 line 22 To interview employed ... omit WITH. | **Study Setting**  To interview employed and non-employed women in the Isfahan metropolis, the researchers will refer to health centers, offices, cultural centers, mosques, parks, recreation centers, clubs, and homes. In this study, participants are selected by purposive sampling. |
|  | p. 8. line 29 phenomena is the plural, form phenomenon is singular | **Study population**  In community-based action research studies, the views of those persons who are at risk should be evaluated. The combination of community members’ experiences with public health science provides a deeper understanding of complex social phenomena Thus, providing more relevant interventions and increasing the likelihood of the interventions can be effective. They can also be adopted, implemented, and sustained in a real-world setting [34]. |
|  | p. 9 line 19 - indent this final para and add a bullet | - Middle-aged Unit of Isfahan Health Center (Middle-aged people health unit plays a role in preventing non-communicable diseases in Isfahan province. This unit operates under the supervision of Isfahan University of Medical Sciences. It attempts to increase life expectancy, reduce the burden of disease and risk factors, and offer public health services with an emphasis on primary care) - Faculty members of Oncology Departments, gynecologists, general surgeons specializing in cancer surgery, adult health nursing, community health nursing, midwifery, and reproductive health in the School of Nursing and Midwifery of Isfahan University of Medical Sciences |
|  | p. 10 line 27 Investigators will use content analysis in this qualitative research project. | **Data analysis method in the qualitative phase**  Investigators will use content analysis in this qualitative research project [36]. In this research, in order to analyse qualitative data, the conventional qualitative content analysis method with Granheim and Landman’s approach is used. First, interviews are copied word-by-word. |
|  | 11.  line 23 - cost eonstraints, AN initial | **1.5. Identification of the barriers and facilitators of the research implementation**  In qualitative research, unlike quantitative research, instead of the number of samples, the quality of the data is emphasized. In qualitative research, the sampling process continues to the point where the researcher does not receive new information from the participants, and only the data should be repeated and verified, even though there is no fixed standard and rule for the number of participants in this kind of research. Given time and cost constraints and initial targeted sample size is usually proposed (eg 30) [37]. |
|  | p. 13. line 44 HEALTH team members | **Data collection**  In the present study, employed and non-employed women of Isfahan metropolis and members of the health group who are eligible are selected using the purposive sampling method. Working women have higher income levels than unemployed women. Therefore, they usually have higher independence of action and their access to public services is higher. The type of job may affect the level of awareness, health beliefs, and access to health services is considered. Thus, the maximum variety of jobs (housewives, workers, faculty members, teachers, health team members, nurses, midwives, doctors, service personnel, government, and non-government administration staff in Isfahan) are taken into account during sampling |
| RESEARCH DESIGN AND METHODS: | p. 14, line 38 A new code ... | Interview and sampling continue until data saturation is reached. Saturation refers to the completion of all categories and to the idea that no new conceptual information that requires a new code or the expansion of new code is obtained. . |
| RESEARCH DESIGN AND METHODS: | lines 46 and 49 FOCUS groups | At the group discussion sessions, the researcher acts as the facilitator and guider of the discussions, and another person is present to take notes. In the present study, the researcher plays the role of the facilitator in focus groups. The role of a facilitator or a moderator is very important in conducting a focus group research. Therefore, it is very important to invite experienced researchers to do such an activity. In the present study, Dr. Savabi is responsible for mediation due to her familiarity and mastery of the subject and breast cancer screening. Also, since focus group is a type of semi-structured group interview, a comprehensive guide is provided to conduct the discussion. This guide is used as a roadmap for group interviews and specifies what goals should be achieved after the group interview. |
| RESEARCH DESIGN AND METHODS: | p. 15 line 15 LEADS to data productionp. | At one end of the spectrum, the mediator plays the role of a co-creator, playing a more active role and intervening more in the debate, while at the other end of the spectrum, the mediator acts only as a data collector and raises only broad and neutral questions and minimizes its role in the debate and intervenes only where it ends leads to data production. |
| RESEARCH DESIGN AND METHODS: | p 16 line 45 agree, CONSENSUS | If more than 80% of the members agree on an area and the quartile range is zero, the agreement is estimated to be very high. If more than 60% of the members agree and the quartile range is more than 1, the agreement is considered moderate; and if less than 60% of the members agree and the quartile range is more than 2, the agreement is considered weak. The consensus is reached when the level of agreement is very high. Therefore, in decision matrices, when more than 80% of members agree, consensus is reached and the strategies are introduced as the suggested ones for self-care behaviors in preventing breast cancer [40]. |
| RESEARCH DESIGN AND METHODS: | p. 21 line 14 - are both sets of references correct? | Yes |
| DISCUSSION | line 24 do you really mean positive symptoms?  or positive sign - what is the environment guide?  I don't understand this phrase | Health belief model constructs consist of perceived sensitivity, perceived intensity, perceived benefits, perceived barriers, self-efficacy, and guidance for action. According to this model, individuals are convinced to carry out disease prevention activities with positive feedback they receive from their environment (ease of doing, being time consuming and also the effective role of self-examination in early diagnosis and treatment of the disease as well as preventing the spread of the disease to other parts of the body and being cost-effectiveness). |
| DISCUSSION | line 28 - lack of trust OF  health .... | In Iranian society, fear of cancer diagnosis [45]. ]., lack of trust of health lack of women’s independence in decision-making, fatefulness, lack of women's empowerment [46]., |
